# Distribution and Availability of Essential Tuberculosis Diagnostic Items in Amhara Region, Ethiopia

**DOI:** 10.1371/journal.pone.0141032

**Published:** 2015-12-07

**Authors:** Mulusew Alemneh Sinishaw, Gebremedhin Berhe Gebregergs, Melashu Balew Shiferaw

**Affiliations:** 1 Bahir Dar Regional Health Research Laboratory Center, Bahir Dar, Ethiopia; 2 School of Public Health, College of Medicine and Health Sciences, Bahir Dar University, Bahir Dar, Ethiopia; Hebrew University, ISRAEL

## Abstract

Adequate supplies of tuberculosis laboratory reagents and consumables are necessary for tuberculosis diagnosis and monitoring of treatment response. This study assessed the distribution and stock levels of laboratory commodities used in tuberculosis control in health centers of Amhara region, Ethiopia. A cross-sectional study was conducted in 82 health centers, among 801, providing sputum microscopy services. Stock levels were calculated, and distribution of reagents and consumables assessed. Thirty three (40.2%) health centers were under stocked for at least one of the key items for tuberculosis diagnosis at the time of visit. Fifteen (18.3%) health centers had no stocks of at least one of the key items (methylene blue (11%), carbol fuchsin (11%), acid alcohol (8.5%) and sputum cups (3.7%)). Of the 82 health centers, 77 (93.9%) did not fulfill the criteria for effective distribution of tuberculosis laboratory reagents and consumables. There were many health centers that had no or only low stocks of key tuberculosis laboratory reagents and consumables as a result of ineffective distribution system. It is necessary to strengthen supply chain management to ensure uninterrupted TB diagnostic service.

## Introduction

Efficient laboratory services are of critical importance in tuberculosis (TB) case detection and monitoring of therapeutic response in bacteriologically confirmed patients [1]. Adequate supplies of TB laboratory reagents and consumables to perform Ziehl-Neelsen staining to detect acid fast bacilli (AFB) are necessary for provision of laboratory services [2].

In 2007, a Logistics Management Information System (LMIS) was introduced by the Ethiopian Federal Ministry of Health to manage the distribution and stock control of laboratory reagents and consumables in the country [3]. The system did not integrate supply chain management of commodities required by different health programs in the country. For instance, Pharmaceuticals and Fund Supply Agency (PFSA) handled the national AIDS programme supplies, including commodities for treatment of opportunistic infections, while the Regional Health Bureaus (RHB) provided TB, malaria, and family planning commodities. This created inefficiencies, duplications of effort, and confusion about responsibilities in the country [3,4].

To solve this situation, the Integrated Pharmaceutical Logistics System (IPLS) was established in 2011 by Federal Ministry of Health. It is meant to strengthen institutional capacity to store and distribute commodities using a single system under the management of one entity: the PFSA [4]. This policy has not been evaluated in Amhara region, Ethiopia. In the region, there are 801 health centers which provide TB laboratory services [5]. In 2013, a total of 28750 TB patients were registered; smear positive, smear negative and extra pulmonary TB was 6453, 9892 and 12225, respectively. From the 6282 enrolled pulmonary TB positive patients, 77.6% were cured[6].The objective of this study was to assess the distribution and stock levels of laboratory reagents and consumables used in TB control in public health centers of Amhara region, Northern Ethiopia.

## Methods

A cross sectional study was undertaken from April 28 to May 26, 2014 in Amhara region. Out of the ten zones in the region, six (South Gondar, North & South Wollo, East & West Gojjam and Oromia) were selected purposively based on geographical representation and accessibility.

TB diagnostic reagents are distributed by regional laboratories to each Zone, Woreda and health center according to their consumption. The consumables are procured from the PFSA directly by health centers or Woreda health offices, and distributed to health centers by PFSA. The regional health bureau supervises the process every six months. This is done to evaluate, support and take corrective actions.

Transportation of consumables to each facility takes place every other month. In case of reagents and emergency orders for consumables, it is a duty of the requesting institution to transport commodities of its order.

**Fig 1:**
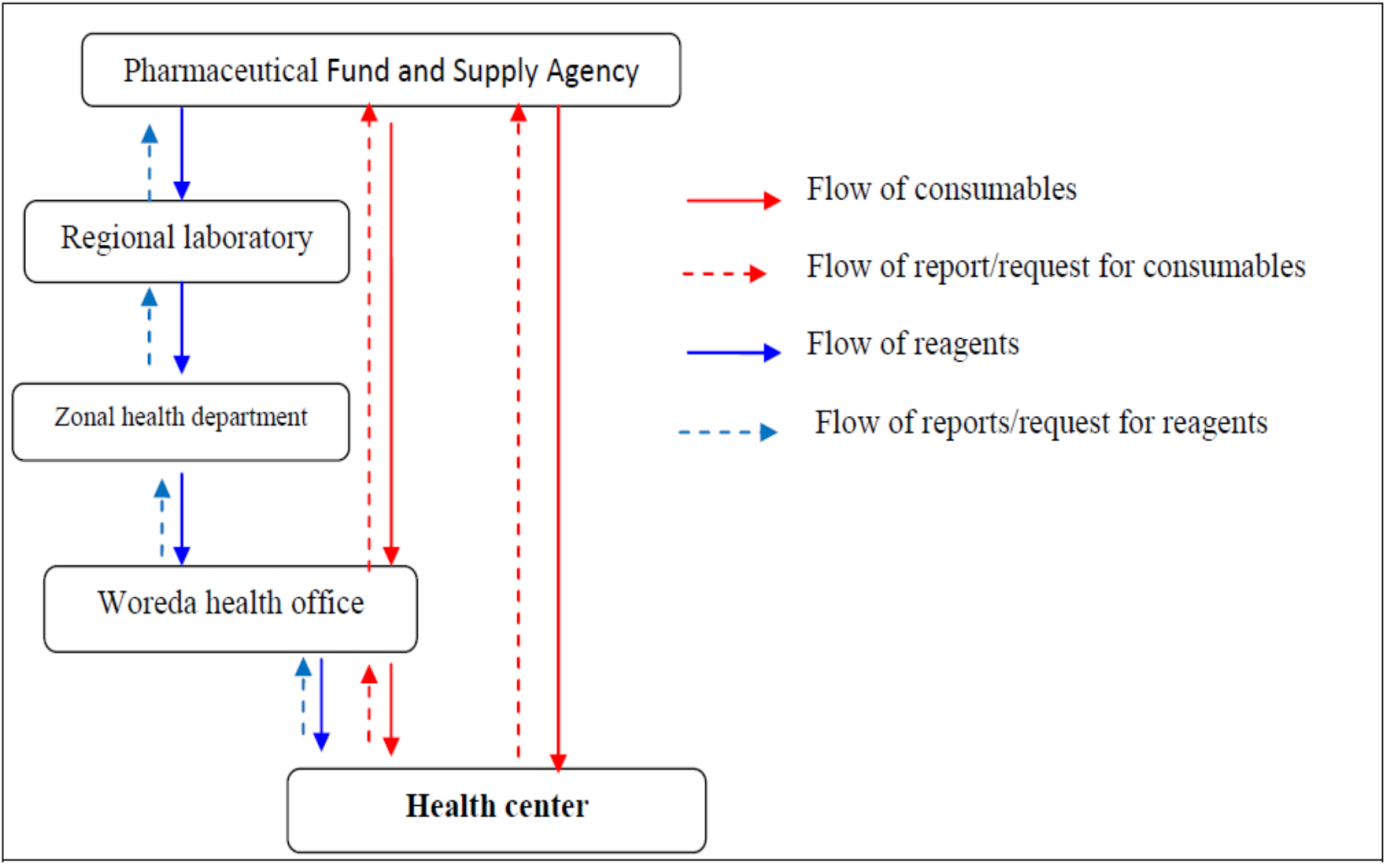
Flow chart showing distribution system of tuberculosis laboratory reagents and consumables in Ethiopia, 2014.

Pharmacy technicians working in the stores use Report and Resupply Form (RRF) to order TB laboratory reagents and consumables from Woreda health office and PFSA. This process is done every other month. The flow of reagents and consumables from the PFSA hub to each service delivery point is illustrated in Fig 1.

All (456) public health centers, in the selected zones, that provided AFB microscopy services and documented their smear results for TB diagnosis from January 01 to March 31, 2014 were included into the study sampling frame. A total of 82 health centers were studied. This sample size was determined by using a single population proportion formula and estimating prevalence of stock outs to be 60.5% [7]. Margin of error was set at 10% with a 95% confidence interval. The zones with the largest number of health centers were allocated the largest number of sampled centers. Health centers in the selected zones were enrolled by simple random sampling using random number generator.

Trained, specifically for this study, laboratory technologists and pharmacists collected data through a review of relevant records that included bin cards, temperature logs and quarterly reports, and physical stock count. They interviewed pharmacy technicians working in the stores and laboratory heads using a pretested questionnaire. The investigators supervised the data collection process.

Double data entry was done using EPI info version 3.5.1 to ensure data quality. Data were analyzed by SPSS version 16. Stock levels and proportions of effective distribution of laboratory reagents and consumables were calculated. Data for one quarter, that is, from January 01 to March 31, 2014, were used to determine stock levels. Each individual health center was the unit of analysis.

The study was approved by the Regional Ethical Review Committee of the Amhara National Regional State Health Bureau. Letter of permission was obtained from the heads of the health centers. Informed verbal consents were obtained from the laboratory heads and pharmacy technicians working in the store using the ethics committee approved consent statements to be interviewed and documented. All the data were coded and accessed by the research team only to assure confidentiality.

### Operational definitions

**Effective distribution system** include inventory control using bin cards (stock cards), not using public transportation to distribute stocks, having appropriate storage, requests based on consumption, clearly labeled reagents and reporting on use every other month.

#### Stock level

Amount of tuberculosis laboratory reagents and consumables available for 4 months (for two months consumption and two months reserve) as calculated based on the previous two months’ consumption.

#### Inventory control

Calculating the required amount, the amount used, the remainder reagents and consumables, and recording expiry date.

#### Immediate response

Management approval of report/request for tuberculosis laboratory reagents and consumables to be availed within two weeks.

#### Key items

These included sputum cups and tuberculosis laboratory reagents (1% carbol fuchsin, 3% acid alcohol and 0.1% methylene blue).

#### Tuberculosis laboratory consumables

Included frosted slides, immersion oil, filter paper, wooden applicator sticks, sputum cups, lens tissue, microscope lens cleaning solution and 95% ethanol.

#### Appropriate storage

Was defined as, a store is clean, free from harmful insects and rodents, had good ceiling, used brown bottles for TB reagents, had good floor, had accessible fire safety extinguisher, shelved TB laboratory reagents and consumables separately from other chemicals.

#### Clear labeling

Was defined as having the name, preparation and expiration dates of the reagent on the container.

#### Reporting on use

**S**ending a report that quantified the amount of consumed tuberculosis laboratory commodities, transferred to other centers, wastage and stocks at hand during the last two months.

#### Requisition of supplies

Was defined as a formula based notification of the quantities of tuberculosis laboratory commodities for four months consumption (two months consumption and two months reserve).

## Results

### Characteristics of study health centers

Of the total study health center laboratories, 81 (98.8%) were linked to the external quality assessment program. Fifty seven (69.5%) health centers exercised health care financing. Almost all (98.2%) health centers had a partner supporting TB activities in terms of: supervision (96.3%), training (96.3%) and provision of consumables (82.9%). Forty two (51.2%) pharmacy technicians in the stores were trained in LMIS in the past two years (Table 1).

**Table 1:** Characteristics of study health centers in Amhara region, 2014. LMIS—Logistic Management Information System, PFSA—Pharmaceuticals and Fund Supply Agency, TB–tuberculosis

Variable	Category	Number	Percent (%)
Pharmacy technicians trained in LMIS	No	40	48.8
	Yes	42	51.2
Laboratory heads trained in LMIS	No	72	87.8
	Yes	10	12.2
Source of TB laboratory consumables	Woreda and PFSA	51	62.2
	Woreda only	23	28.0
	PFSA only	8	9.8

Pharmacy technicians working in the stores used report and resupply form (the standard) in 78 (95.1%) health centers to order TB laboratory reagents and consumables from Woreda health office and PFSA. In 80 (97.6%) health center stores, pharmacy technicians did not monitor room temperatures. A fire safety extinguisher was unavailable in 69 (84.1%) health centers (Table 2).

**Table 2:** Condition of health center pharmacy stores in Amhara region, 2014.

Variable	Category	Number	%
Free from harmful insects and rodents	No	8	9.8
	Yes	74	90.2
Good ceiling (avoid sunlight and water leakage)	No	6	7.3
	Yes	76	92.7
Brown bottles used for tuberculosis reagents	No	9	11.0
	Yes	73	89.0
Good floor (no contact with water and humidity)	No	0	0.0
	Yes	82	100.0
Fire safety extinguisher was available and accessible	No	69	84.1
	Yes	13	15.9
TB laboratory commodities were separately stored from insecticides and chemicals	No	26	31.7
	Yes	56	68.3
Store room was clean (all trash removed, sturdy shelves and organized boxes)	No	8	9.8
	Yes	74	90.2

### Availability of TB laboratory reagents and consumables

**Fig 2:**
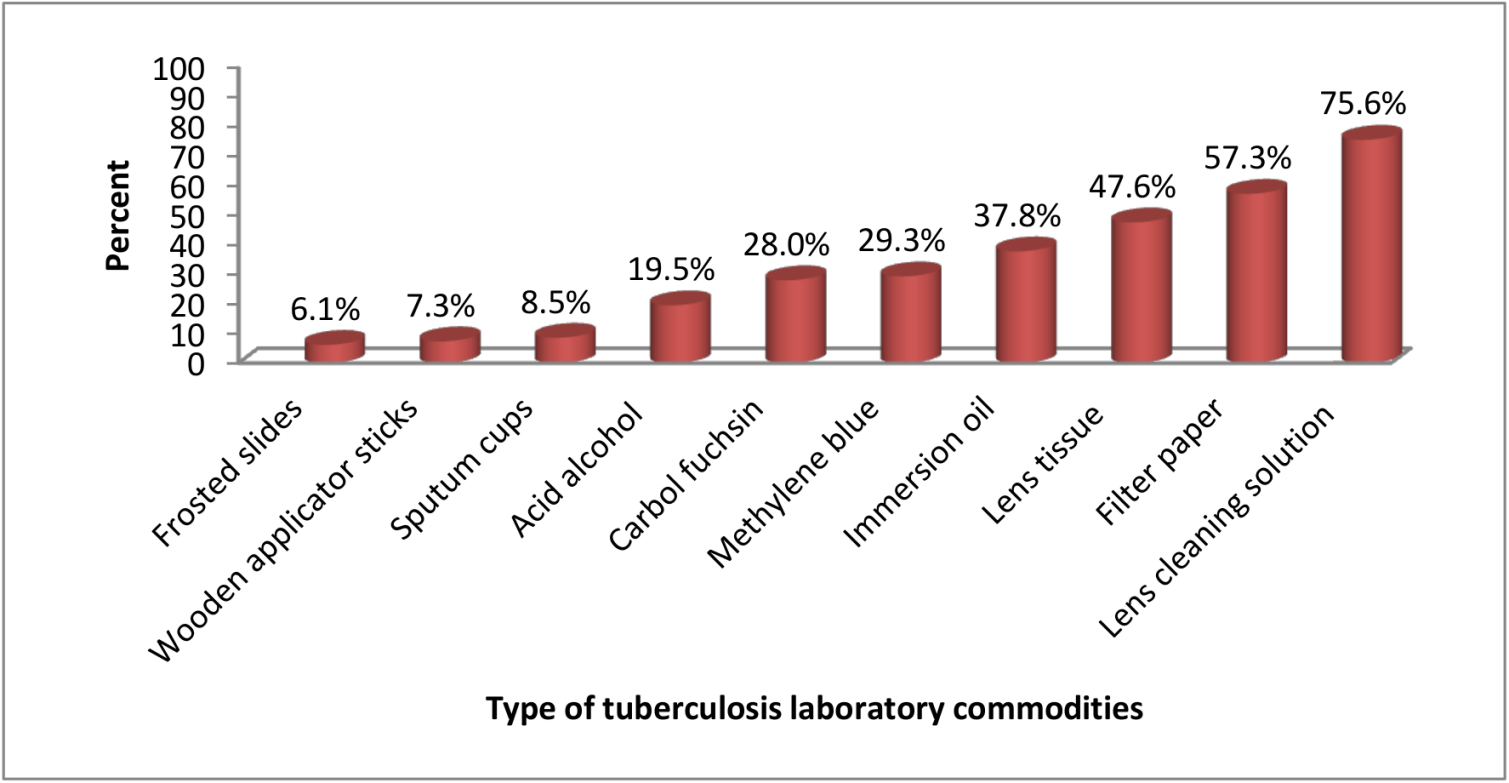
Under stock of tuberculosis laboratory commodities at health centers in Amhara region, 2014.

Thirty three (40.2%) health centers were under stocked for at least one of the key items. Eleven (13.4%) health centers were under stocked for all TB diagnostic reagents (Fig 2).

Fifteen (18.3%) health centers had a complete stock out of at least one of the key items (methylene blue 9 (11.0%), carbol fuchsin 9 (11.0%), acid alcohol 7 (8.5%) and sputum cups 3 (3.7%). Three health centers had a complete stock out of all TB diagnostic reagents.

### Distribution of TB laboratory reagents and consumables

**Fig 3:**
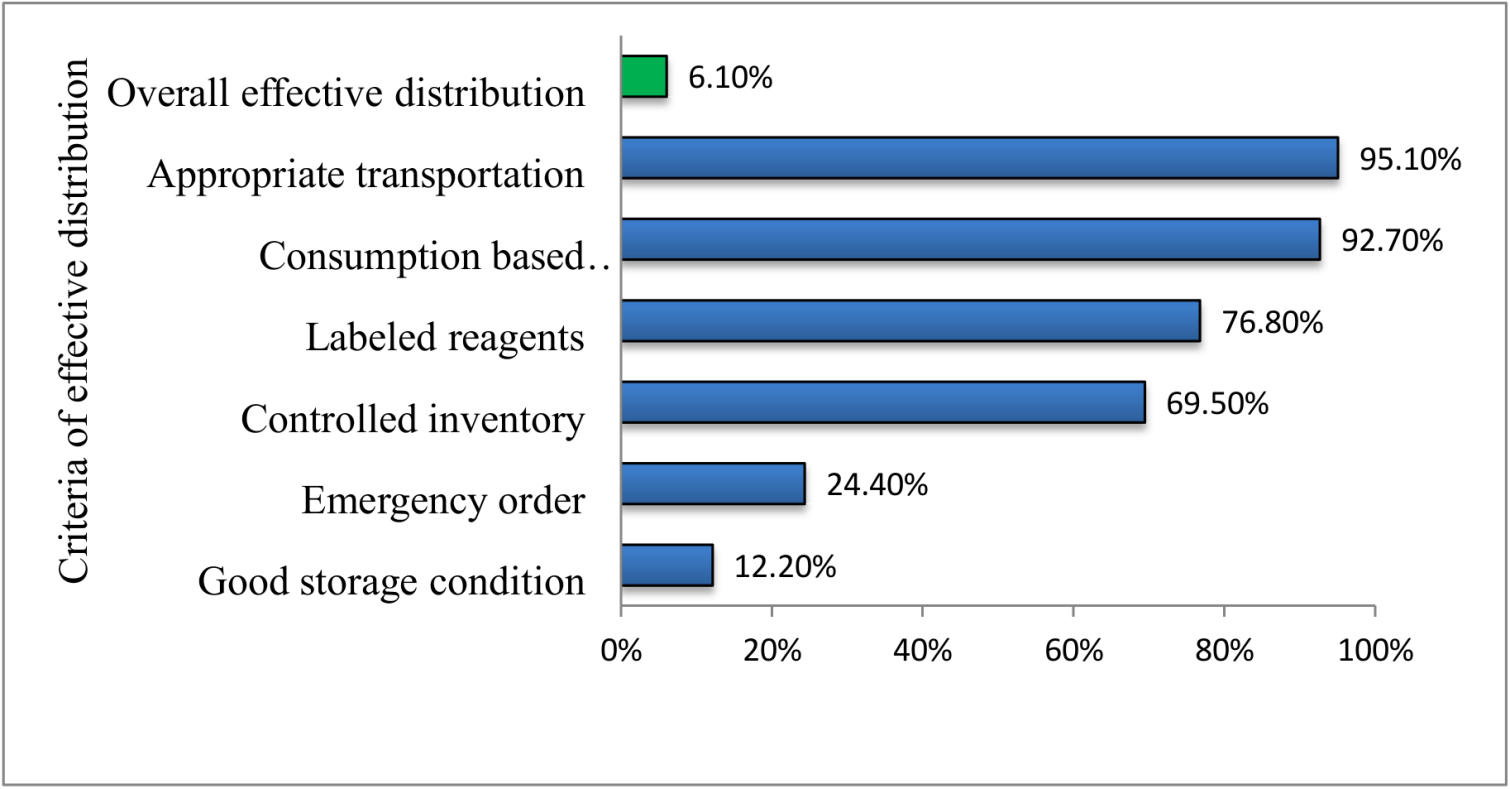
Distribution criteria for tuberculosis diagnostic reagents and consumables in Amhara region, 2014.

In this study, 77 (93.9%) health centers did not fulfill the criteria for effective distribution of TB laboratory reagents and consumables. Seventy six (92.7%) health centers sent reports or orders for TB laboratory reagents and consumables to the higher level every 2 months that meets the standard. Of these, thirty six (47.4%) health centers obtained the requested quantity; Fig 3.

## Discussion

In management of TB control services, stock outs of TB laboratory commodities are unacceptable. All TB diagnostic laboratory commodities are essential for diagnosis and follow-up tests of TB patients, and must be available at all times [8]. However, in our study we found that four out of ten health centers were under stocked and 18% had a complete stock out of at least one of the key items. This is not a problem of the region only. Nationally, it has been stated that supplies interruptions (stock outs of laboratory reagents) is one of major challenges of TB Control in Ethiopia [9].

Low stock levels or no supplies could be due to reluctance of health personnel at all levels and supplies might have been run out in the PFSA hubs as only 36 (47.4%) health centers obtained the requested quantity. In addition, one of four health managers did not approve to avail the requested TB laboratory reagents and consumables within 2 weeks as set by the standard. In developing countries where public agency is responsible for procurement of health commodities often lack technical capacity to efficiently ensure supply security [10].

Carbol fuchsin, methylene blue and acid alcohol were out of stock in 11%, 11% and 8.5% health centers, respectively, at the time of visit. A study in Addis Ababa showed that only 2.9% of facilities had no stocks of acid alcohol and all had adequate supplies of carbon fuchsin and methylene blue on the day of visit [7]. In Malawi, 5% of health centers had stock outs of carbon fuchsin, methylene blue and acid alcohol [11]. The higher stock out rate found in our study could be due to the rural setting where distances are long and transportation poses challenges. The higher sample size in this study as compared to the previous ones might have included heterogeneous health centers that contribute for the difference. Generally, health facilities and health care providers cannot offer services without the appropriate health commodities [12].

In this study, most health centers did not fulfill the criteria for effective distribution of TB laboratory reagents and consumables. This poses a great challenge to the sustainable supply of TB laboratory reagents and consumables which is likely to be associated with weak TB diagnostic and follow up services. Frail supply chain management could also contribute to delays in detection of active TB disease and even to a low case finding rate. Delayed approval processes, weaknesses in procurement and distribution systems were reported in Nigeria [13]. Overall, procurement and distribution systems of TB commodities and equipment in developing countries have been described as weak, erratic and dysfunctional [14].

Nine out of ten health centers had no appropriate storage for TB laboratory reagents and consumables. Reagents and consumables were found to be directly exposed to sun light, they were stored mixed with other chemicals and store room temperatures were not monitored. In line with our findings, in Angola, warehouses had low ceilings making them warm and congested [15]. Warehouse temperatures should be controlled and monitored and appropriate action should be taken if temperatures exceed the recommended level [16].

The main study limitations included small sample size and the fact that laboratories in the remote zones of the region were not surveyed. It follows that the study findings may not be generalized to the entire Amhara region.

## Conclusion

There were many health centers that had no or low stocks of key TB laboratory reagents and consumables as a result of an ineffective distribution system. It is necessary to strengthen supply chain management to ensure uninterrupted TB diagnostic services.

## Supporting Information

S1 Data SPSS data. (SAV)

S1 Text Questionnaire (data collection tool). (DOCX)
