# Supplementary material for: Distribution and Availability of Essential Tuberculosis Diagnostic Items in Amhara Region, Ethiopia
Source: PLoS One. 2015 Dec 7;10(12):e0141032. doi: 10.1371/journal.pone.0141032 (PMC4671647; doi:10.1371/journal.pone.0141032)
Supplement: S1 Text — (DOCX) [file pone.0141032.s002.docx]

# Data collection tool

## Information sheet and consent form

**Information sheet**

**Title of the research**: Assessments of Distribution and availability of TB laboratory commodities used for Diagnosis of Tuberculosis in Public Health Centers of Amhara Region, North Ethiopia.

**Name of Principal Investigator**: Mulusew Alemneh Sinishaw.

**Name of the Organization:** Bahir Dar Regional Health Research lab center

**Sponsor for the Project፡**: TB care/ USAID through Federal Ministry of Health TRAC

**Purpose of the Research Project**

The aim of this study is to assess whether Distribution and availability of TB laboratory commodities used for Diagnosis of Tuberculosis is up to standard in Public Health Centers of Amhara Region, Ethiopia.

**Procedure**

Your health center is chosen by chance and we are inviting you to take part in the study. Your participation will help us to assess the level of comprehensive Distribution and availability of TB laboratory commodities used for Diagnosis of Tuberculosis in Public Health Centers of Amhara Region, North Ethiopia. We are going to ask you some questions that are not difficult to answer. Your honest answers are very important to our study. We are also going to review quarterly reports, models, and bin cards in the health center. We would like to appreciate your help in responding to these questions.

**Benefit**:

When you are participating in this research, there may not be a direct benefit to you. However, your participation is very indispensible to inform us about potential problems related to TB lab commodities supply in public health centers of Amhara region and will assist us to design and deliver uninterrupted supply for it.

**Risk and/or Discomfort**

There is no risk when you are participating in this research project except using part of your time (about 30 minutes).

**Confidentiality**

The information collected for this research project will be kept secured and your name will not be written in the forms or appear in the database and never be used in connection with any of the information you are going to give.

**Right to Refusal or Withdraw**.

You have the right to refuse participating in this research and you are not obliged to answer any question that you do not want to answer and you may end it at any time you want to.

Your participation will not affect your position or career in the health center you are working.

**Contact Person**

If you have any question you can contact the following individuals.

Wendimu Gebeyehu (Chair person for Amhara regional ethical review committee and head of Bahir Dar Regional health research laboratory center), Mobile: +251918780270 / e-mail wgebeyehu61@gmail.com

Mulusew Alemneh (Principal investigator) Mobile: +251921280734 / e-mail mulusewalemneh@yahoo.com

**Consent statement**

Dear Respondent: I am __________________________________. I am here to collect information on this health center TB lab commodities supply. Do you agree to participate in the study by responding to the questions already prepared?

1.       Yes     2.  No

Thank you for your response!

## Questionnaire

General instruction: this questionnaire will be delivered to each public health center to assess the comprehensive Distribution and availability of TB laboratory commodities used for Diagnosis of Tuberculosis in Public Health Centers of Amhara Region, North Ethiopia.

Zone: ______________ Woreda: ____________ Kebele: _______________

Health facility code No: _______________ Date of visit: ________________

### Part IA: Interview questions for pharmacy store man

Qualification of pharmacy personnel working in the store___________________

| **S No** | **Questions** | | **Categories and Codes** | **Skip** |
| --- | --- | --- | --- | --- |
| 1 | From where do you get TB lab consumables? | | 1. Woreda health office  2. PFSA  3.Both  4. Other (specify) __________________ |  |
| 2 | How often are reports or orders for TB lab commodities sent to the higher level (PFSA/Woreda)?  *(This is on one sheet of paper if they use the standard format)* | | 1. monthly  2. every two month  3. Quarterly  4. Every 6 month |  |
| 3 | How does your health center determine how much of TB laboratory commodities to order? | | 1. formula  2. based on current stock levels  3. do not know  4. others (specify)-------__________________ |  |
| 3.1 | If it was based on formula observe the document and categorize the type? | | 1. performance calculation  2. IFRR |  |
| 4 | Is the health center using a health care financing system? | | 1. No 2. Yes |  |
| 5 | Did you get TB lab commodities from supplier agencies (PFSA) as ordered amount? | | 1. No  2. Yes |  |
| 6 | Did this health center use an emergency order for TB lab commodities in the last quarter? | | 1. No  2. Yes |  |
| 7 | What forms do you use for reporting/ordering TB laboratory commodities? (Observe their use) | | | |
| 7.1 | Government receiving note (Model 19) | 1. No 2. Yes | |  |
| 7.2 | Facility supplies report and resupply format (RRF) | 1. No 2. Yes | |  |
| 7.3 | Internal Facility report and resupply (IFRR) | 1. No 2. Yes | |  |
| 8 | Did health center management avail TB laboratory commodities within two weeks of request? | 1.No  2Yes | |  |
| 9 | Do you follow your balance for TB lab commodities using bin cards? | 1. No  2. Yes (if yes check by observing from the bin card) | |  |
| 9.1 | Is the expiration dates for reagents and supplies recorded by using bin card? | 1. No  2. Yes (if yes check by observing from the bin card) | |  |
| 10 | Is there visually clear labeling (name, manufacturing (preparation) date and expiration date on reagents container? | 1. No  2. Yes (observe) | |  |
| 11 | Are TB lab commodities stored in a manner accessible for first expire first out (FEFO), counting and management? | 1. No  2. Yes (observe!) | |  |
| 12 | Do you get training on logistic management information system in the last two years? | 1. No  2. Yes | |  |
| **13** | **The following two questions address storage condition of the room** | | | |
| 13.1 | Do you monitor your store temperature regularly (twice a day)? | | 1. No  2. Yes (if yes check by observing temperature record) | If no go to 12.3 |
| 13.2 | If the answer for 20.1 is yes record the minimum and maximum value of room temperature. | | Minimum T^o^_____ ^o^C  Maximum T^o^_____ ^o^C |  |
| 13.3 | If the answer for 20.1 is no, the reason not to monitoring is_____________ | | ______________________________________________________ |  |
| 13.4 | Do you use brown bottle for reagents? | | 1. No  2. Yes (if yes check by observing it) |  |
| 13.5 | are TB lab commodities protected from water and humidity | | 1. No 2. Yes |  |
| **MAKE OBSERVATION IN THE STORE ROOM TO ADRESS THE FOLLOWING QUESTIONS:** | | | | |
| 13.6 | Storage area is visually free from harmful insects and rodents (check for droppings and/or insects) | | 1. No  2. Yes |  |
| 13.7 | Roof is maintained in good condition to avoid sunlight and water penetration | | 1. No  2. Yes |  |
| 13.8 | Fire safety extinguisher is available and accessible | | 1. No 2. Yes |  |
| 13.9 | TB lab commodities are stored separately from insecticides and other chemicals | | 1. No  2. Yes |  |
| 13.10 | Store room is clean (all trashes removed, sturdy shelves and organized box? | | 1. No  2. Yes |  |
| 14 | Have there been unexplained losses (wastage)? | | 1. No  2. Yes(if yes check from the bin card) |  |
| 15. ANY OTHER OBSERVATIONS MADE: | | | | |

Thank you for your time and information. You have been very helpful. Our remaining question will require looking at products in the storeroom.

### Part I B: Check list for supplies at pharmacy store and lab mini-store

| S.  No | Type of supplies | Availability ( if tick on the space under each choice) | | | | Where do you get your supply? | | Remark |
| --- | --- | --- | --- | --- | --- | --- | --- | --- |
|  |  | No | Yes | If yes write | | Woreda | Purchasing |  |
|  |  |  |  | Unit | quantity |  |  |  |
| 1 | 1% carbol fuchsin |  |  |  |  |  |  |  |
| 2 | 3% acid alcohol |  |  |  |  |  |  |  |
| 3 | 0.1% methylene blue |  |  |  |  |  |  |  |
| 4 | 95% of ethanol for heating carbol fuchsin |  |  |  |  |  |  |  |
| 5 | Sputum cups |  |  |  |  |  |  |  |
| 6 | Reagent bottle |  |  |  |  |  |  |  |
| 7 | Dispensing bottle |  |  |  |  |  |  |  |
| 8 | Frosted slides |  |  |  |  |  |  |  |
| 9 | wooden applicator stick/wire loop |  |  |  |  |  |  |  |
| 10 | Funnel for reagents |  |  |  |  |  |  |  |
| 11 | Filter paper |  |  |  |  |  |  |  |
| 12 | Lens tissue/ lent free soft |  |  |  |  |  |  |  |
| 13 | Oil immersion |  |  |  |  |  |  |  |
| 14 | Microscope lens cleaning solution(di-ethyl/alcohol) |  |  |  |  |  |  |  |

### Part IIA. Interview questions for Lab Head

| **S. No** | **Questions** | **Categories and Codes** | | **Skip** |
| --- | --- | --- | --- | --- |
| 1 | Is there any partner support for TB or TB/HIV diagnosis? | 1. No  2. Yes | | **If no go to Q. 3** |
| 1.1 | If the answer is yes for Q. 2 when they start to support? | ______________ | |  |
| 1.2 | If the answer is yes for Q. 2 what kind of support they give? | 1. Training  2. Consumables  3. Supportive supervision  4. Others (specify) ____________________________________ | |  |
| 2 | Is your lab EQA networked? | 1. No 2. Yes | |  |
| 3 | Did you participate /give specification for purchasing of TB lab commodities for the health center to give for procurement officers? | 1. No 2. Yes | | If yes skip to Q. 5 |
| 3.1 | If the answer is no for Q.4 what is your reason not to participate? | ___________________________________________________ | |  |
| 4 | Do you request supplies based on report of your use? | 1. No  2. Yes(if yes observe the recent two weeks or quarterly report) | |  |
| 4.1 | If it was based on formula observe the document and categorize the type? | 1. performance calculation  2. IFRR | |  |
| 4.2 | If “No” for Q. 5, how does your health center determine how much of TB laboratory commodities to order? | 1. based on current stock levels  2. do not know  3. others (specify) _________________ | |  |
| 5 | Total number of patients diagnosed for TB in the previous quarter (2 months used for refill calculation)? | New (_______)  Follow up (_______)  Total slides (______) | |  |
| 6 | Do you have mini-store for this lab? | 1. No  2. Yes | If yes continue for the next questions | |
| 7 | Do you follow your stock balance for reagents and supplies by using bin cards? | 1. No  2. Yes(if yes observe from the bin card) | |  |
| 7.1 | Is there proper labeling (name, manufacturing (preparation) date and expiration date) on reagents container? | 1. No  2. Yes | |  |
| 7.2 | Is the expiration dates for reagents and supplies recorded by using bin card? | 1. No  2. Yes(if yes observe bin card) | |  |
| 8 | The following four questions address storage condition of the room | | | |
| 8.1 | Did you monitor your store temperature regularly (twice a day)? | 1. No  2. Yes(if yes observe temperature record) | | If no go to 9.3 |
| 8.2 | If the answer for 9.1 is yes record the minimum and maximum value of room temperature. | Minimum T^o^_____ ^o^C  Maximum T^o^____ ^o^C | |  |
| 8.3 | If the answer for 9.1 is no, the reason not to monitoring is_____________ | ______________________________________________________ | |  |
| 8.4 | Do you use brown bottle for reagents? | 1. No  2. Yes(if yes observe it) | |  |
| 9 | Have there been unexplained losses (wastage)? | 1. No  2. Yes(if yes it from bin card) | |  |
| 10 | Do you get training on logistic management information system in the last two years? | 1. No  2. Yes | |  |

### Part II B: Check list for supplies at lab mini-store or at lab

| S.  No | Type of supplies | Availability ( if tick on the space under each choice) | | | | Where do you get your supply? | | Remark |
| --- | --- | --- | --- | --- | --- | --- | --- | --- |
|  |  | No | Yes | If yes write | | Woreda | Purchasing |  |
|  |  |  |  | Unit | quantity |  |  |  |
| 1 | 1% carbol fuchsin |  |  |  |  |  |  |  |
| 2 | 3% acid alcohol |  |  |  |  |  |  |  |
| 3 | 0.1% methylene blue |  |  |  |  |  |  |  |
| 4 | 95% of ethanol for heating carbol fuchsin |  |  |  |  |  |  |  |
| 5 | Sputum cups |  |  |  |  |  |  |  |
| 6 | Reagent bottle |  |  |  |  |  |  |  |
| 7 | Dispensing bottle |  |  |  |  |  |  |  |
| 8 | Frosted slides |  |  |  |  |  |  |  |
| 9 | wooden applicator stick/wire loop |  |  |  |  |  |  |  |
| 10 | Funnel for reagents |  |  |  |  |  |  |  |
| 11 | Filter paper |  |  |  |  |  |  |  |
| 12 | Lens tissue/ lent free soft |  |  |  |  |  |  |  |
| 13 | Oil immersion |  |  |  |  |  |  |  |
| 14 | Microscope lens cleaning solution(di-ethyl/alcohol) |  |  |  |  |  |  |  |
